# Efficacy and safety of neoadjuvant FOLFIRINOX for borderline resectable pancreatic adenocarcinoma: improved efficacy compared with gemcitabine-based regimen

**DOI:** 10.18632/oncotarget.17940

**Published:** 2017-05-16

**Authors:** Changhoon Yoo, Jihoon Kang, Kyu-Pyo Kim, Jae-Lyun Lee, Baek-Yeol Ryoo, Heung-Moon Chang, Sang Soo Lee, Do Hyun Park, Tae Jun Song, Dong Wan Seo, Sung Koo Lee, Myung-Hwan Kim, Jin-Hong Park, Dae Wook Hwang, Ki Byung Song, Jae Hoon Lee, Song Cheol Kim

**Affiliations:** ^1^ Department of Oncology, Asan Medical Center, University of Ulsan College of Medicine, Seoul, Korea; ^2^ Department of Gastroenterology, Asan Medical Center, University of Ulsan College of Medicine, Seoul, Korea; ^3^ Department of Radiation Oncology, Asan Medical Center, University of Ulsan College of Medicine, Seoul, Korea; ^4^ Department of Surgery, Asan Medical Center, University of Ulsan College of Medicine, Seoul, Korea

**Keywords:** pancreatic cancer, chemotherapy, neoadjuvant, FOLFIRINOX

## Abstract

Borderline resectable pancreatic cancer (BRPC) is a potentially resectable disease but is associated with poorer survival compared to primary resectable disease. There has been no prospective trial that compare the efficacy of FOLFIRNOX and gemcitabine-based regimen for BRPC. Between February 2013 and December 2014, 18 patients with BRPC receiving FOLFIRINOX were reviewed retrospectively. For comparative analysis, data for all BRPC patients (n=18) in our previous phase 2 study of neoadjuvant fixed-dose rate-gemcitabine plus capecitabine were pooled. Patients received a median 6 cycles (range, 3-13) of FOLFIRINOX. Surgical resection was performed in 12 patients (67%) and R0 resection in 9 patients. Median progression-free survival (PFS) and overall survival (OS) were 16.8 (95% confidence interval [CI], 9.4-24.2) and 21.2 (95% CI, 14.2-28.2) months, respectively. Patients who underwent surgical resection showed significantly better PFS (p=0.01) and OS (p=0.003) than those unresected. In the exploratory analysis, patients receiving FOLFIRINOX showed significantly longer PFS compared to those receiving fixed-dose rate-gemcitabine plus capecitabine (median 16.8 months [95% CI, 9.4-24.2] vs. 6.5 months [1.6-11.3]; p = 0.04). There was a trend toward improved OS in patients who received FOLFIRINOX (median 21.2 months [95% CI, 14.2-28.2]) compared to those who received fixed-dose rate-gemcitabine plus capecitabine (13.6 months [11.^8^–^15^.^4^]; p=0.12). FOLFIRINOX was feasible and effective as neoadjuvant chemotherapy for patients with BRPC and may have improved efficacy compared to a gemcitabine-based regimen.

## INTRODUCTION

Pancreatic adenocarcinoma is a well-known disease with poor prognosis, having a 5-year survival rate of less than 6% [1]. At the time of diagnosis, 10%–20% of patients with pancreatic cancer are categorized with disease that is potentially curable with surgical resection [2].

Locally advanced, non-metastatic pancreatic cancer (LAPC) occurs in about 30% of newly diagnosed patients [2]. The resectability of pancreatic cancer is mostly determined by the tumor extent in the context of invasion into the celiac artery, superior mesenteric artery/vein, portal vein, and hepatic artery. Recently, the entity of LAPC has been subdivided into borderline resectable and non-resectable disease [3]. Compared to primarily resectable or unresectable disease, borderline resectable pancreatic cancer (BRPC) can be defined as potentially resectable disease with a higher risk of positive resection margins, indicating poorer survival outcomes compared with primarily resectable disease. The criteria most commonly used to define BRPC are suggested by the National Comprehensive Cancer Network (NCCN) [4] and the joint consensus conference of the Americas Hepato-Pancreato-Biliary Association (AHPBA), the Society of Surgical Oncology (SSO), and the Society for Surgery of the Alimentary Tract (SSAT) [5], although some centers have their own criteria based on multidisciplinary board review.

Although surgical resection can be primarily performed for patients with BRPC, neoadjuvant therapeutic strategies have been widely investigated for this disease entity [6], because of the higher chances of margin-positive status and poorer prognosis compared with resectable pancreatic cancer. In recent years, the efficacy of systemic chemotherapy for pancreatic cancer has been enhanced using combination chemotherapy regimens such as FOLFIRINOX (fluorouracil [5-FU], leucovorin, irinotecan, and oxaliplatin) and gemcitabine plus *nab*-paclitaxel [7, 8]. Because these regimens have been primarily tested in trials for patients with metastatic pancreatic adenocarcinoma, however, the efficacy of these regimens in LAPC has not been clearly demonstrated. FOLFIRINOX is now globally accepted as the standard first-line chemotherapy for metastatic pancreatic cancer based on the success of the PRODIGY 4/ACCORD 11 trial, which showed a significantly improved OS of 11.1 months with FOLFIRINOX compared to 6.8 months with gemcitabine [7]. Although many studies have investigated the role of FOLFIRINOX in LAPC, most of these are retrospective analyses and based on small sample sizes, which limit the ability to draw any definitive conclusions regarding the use of FOLFIRINOX in LAPC [6]. Moreover, comparisons of the results across the studies of neoadjuvant chemotherapy are difficult because most studies included both BRPC and unresectable disease [6].

Considering the distinctive clinical behavior and outcomes of BRPC and unresectable locally advanced pancreatic cancer, data regarding neoadjuvant chemotherapy should be presented separately to allow better interpretation and comparisons between trials [3]. Therefore, we retrospectively analyzed the outcomes of neoadjuvant FOLFIRINOX for patients with BRPC only. In addition, there has been no comparative analysis between FOLFIRINOX and gemcitabine-based regimens for BRPC. We performed exploratory analyses to compare the efficacy of FOLFIRINOX and a gemcitabine-based regimen with pooled analysis of our previous neoadjuvant chemotherapy trial [9].

## RESULTS

### Patient characteristics

Baseline characteristics of BRPC patients treated with neoadjuvant FOLFIRINOX are summarized in Table 1. The median age was 54 years (range, 29–73 years) and 9 patients (50%) were men. The most frequent primary tumor site was the head of the pancreas (n = 10, 55%). Baseline cancer antigen 19-9 (CA 19-9) was elevated in 14 patients (78%).

**Table 1:** Baseline patient characteristics

	No (%)
**Sex**	
Male	9 (50%)
Female	9 (50%)
**Age, median**	54 (range, 29–73)
**Primary tumor site**	
Head	10 (55%)
Body or tail	7 (39%)
Multicentric	1 (6%)
**Baseline CA19-9**	
Normal	4 (22%)
>1 & ≤ 2 x UNL	4 (22%)
>2 x UNL	10 (56%)
**Regional lymph node metastasis**	10 (56%)
**No. of chemotherapy cycles**	6 (range, 3–13)
**Surgery**	12 (67%)
R0	9 (75%)
R1	3 (25%)
**Postoperative treatment (n = 12)**	
No	2 (17%)
Chemotherapy only	7 (58%)
CCRT only	2 (17%)
CCRT followed by chemotherapy	1 (8%)
**Postop chemotherapy regimen**	
Gemcitabine	3 (38%)
FOLFIRINOX	5 (62%)

UNL: upper normal limit; CCRT: concurrent chemoradiotherapy.

A median of 6 cycles (range, 3–13 cycles) of neoadjuvant FOLFIRINOX were administered, and the treatment was discontinued because of surgery (n = 12, 67%); change of treatment due to the inadequate tumor response for curative resection with FOLFIRINOX in the status of stable disease (n = 4, 22%); toxicities (n = 1, 6%); and disease progression after initially stable disease (n = 1, 6%). In 6 patients who discontinued FOLFIRINOX due to causes other than surgery, 3 (50%) patients received concurrent chemoradiotherapy (CCRT) and 2 (40%) patients were treated with stereotactic body radiotherapy (SBRT) followed by gemcitabine chemotherapy; and 1 patient who progressed on FOLFIRINOX received gemcitabine plus erlotinib as second-line treatment.

### Safety of neoadjuvant FOLFIRINOX

The mean relative dose intensity (RDI; the total delivered dose as a percentage of the targeted dose per unit time) of each drug during the first four cycles of FOLFIRINOX was as follows: 85% for infusional 5-FU, 84% for oxaliplatin, and 78% for irinotecan (Supplementary Figure 3). The RDI during the subsequent four cycles was maintained at 80% for infusional 5-FU, 80% for oxaliplatin, and 73% for irinotecan. No patient received primary or secondary granulocyte-colony stimulating factor (G-CSF) prophylaxis.

Adverse events associated with neoadjuvant FOLFIRINOX are summarized in Table 2. There was no treatment-related mortality. The most common grade 3–4 adverse events were neutropenia (83%), nausea (33%), fatigue (6%), and febrile neutropenia (6%). No grade 4 non-hematologic toxicities were observed. FOLFIRINOX doses were reduced in 14 (78%) patients, and the most common causes of dose modifications were neutropenia (79%) and nausea or vomiting (21%).

**Table 2:** Adverse events with neoadjuvant FOLFIRINOX

	Any grade (%)	Grade 3–4 (%)
Anemia	16 (89%)	0
Leukopenia	15 (83%)	4 (22%)
Neutropenia	16 (89%)	15 (83%)
Thrombocytopenia	12 (67%)	0
Febrile neutropenia	1 (6%)	1 (6%)
Fatigue	6 (33%)	1 (6%)
Vomiting	5 (28%)	2 (11%)
Nausea	8 (44%)	6 (33%)
Abdominal pain	5 (28%)	0
Diarrhea	8 (6%)	1 (6%)
Hyperbilirubinemia	4 (22%)	0
Elevated liver enzymes	10 (56%)	0
Anorexia	4 (22%)	0
Constipation	0	0
Peripheral neuropathy	2 (11%)	0

### Efficacy of neoadjuvant FOLFIRINOX

Partial response was achieved with neoadjuvant FOLFIRINOX in 6 patients (33%) and stable disease in 12 patients (67%). Surgical resection was performed in 12 patients, indicating a 66.7% resection rate after a median of 6 cycles of FOLFIRINOX (range, 4–13 cycles); R0 and R1 resection were achieved in 9 (75%) and 3 patients (25%), respectively. There were no patients with macroscopic residual disease (R2 resection). Vascular resections were performed in 9 patients (75%); the portal vein, celiac axis, superior mesenteric vein, and hepatic artery were resected in 4 (33%), 3 (25%), 2 (17%), and 2 (17%) patients, respectively. The post-treatment pathologic stage according to the American Joint Committee on Caner (AJCC) 7^th^ edition was IIA in 7 patients (58%), IIB in 4 (33%), and III in 1 (8%). No patients achieved pathologic complete response. According to the College of American Pathologists (CAP) grading protocol [10], 5 (42%) and 7 (58%) patients were classified as grade 2 (moderate response; residual cancer outgrown by fibrosis) and grade 3 (poor or no response; extensive residual cancer), respectively. Following surgery, adjuvant chemotherapy and/or CCRT were performed in all but 2 patients (83%); 7 (58%) patients received chemotherapy alone (FOLFIRINOX in 4 [57%] and gemcitabine in 3 [43%]), 2 (17%) patients received CCRT alone and 1 (8%) received CCRT followed by chemotherapy (FOLFIRINOX). Adjuvant CCRT was performed for patients with margin-positive resection.

Among the patients who underwent surgery, 6 (50%) patients had experienced recurrence at the time of analysis. The most common sites for recurrence after surgery were liver, peritoneal, and locoregional lesions (2 patients each, 33%).

**Figure 1:**
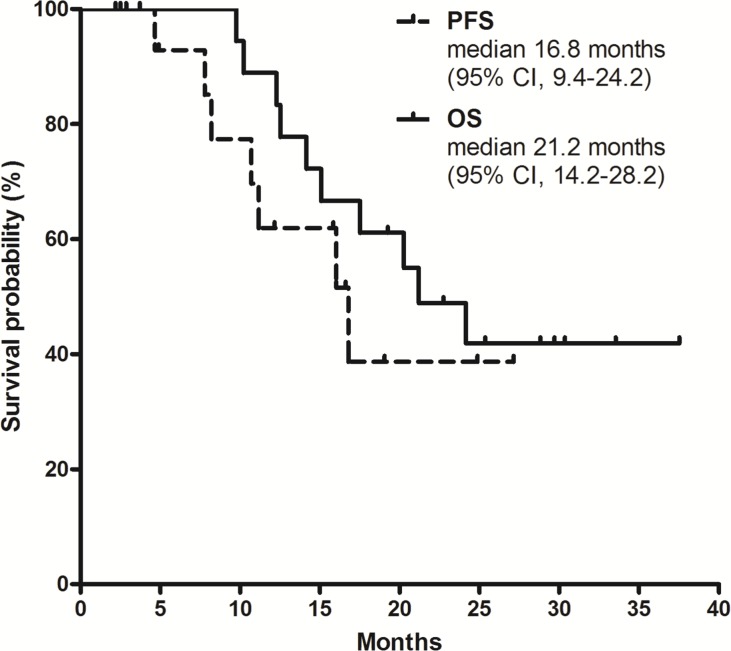
Survival outcomes with FOLFIRINOX

**Figure 2:**
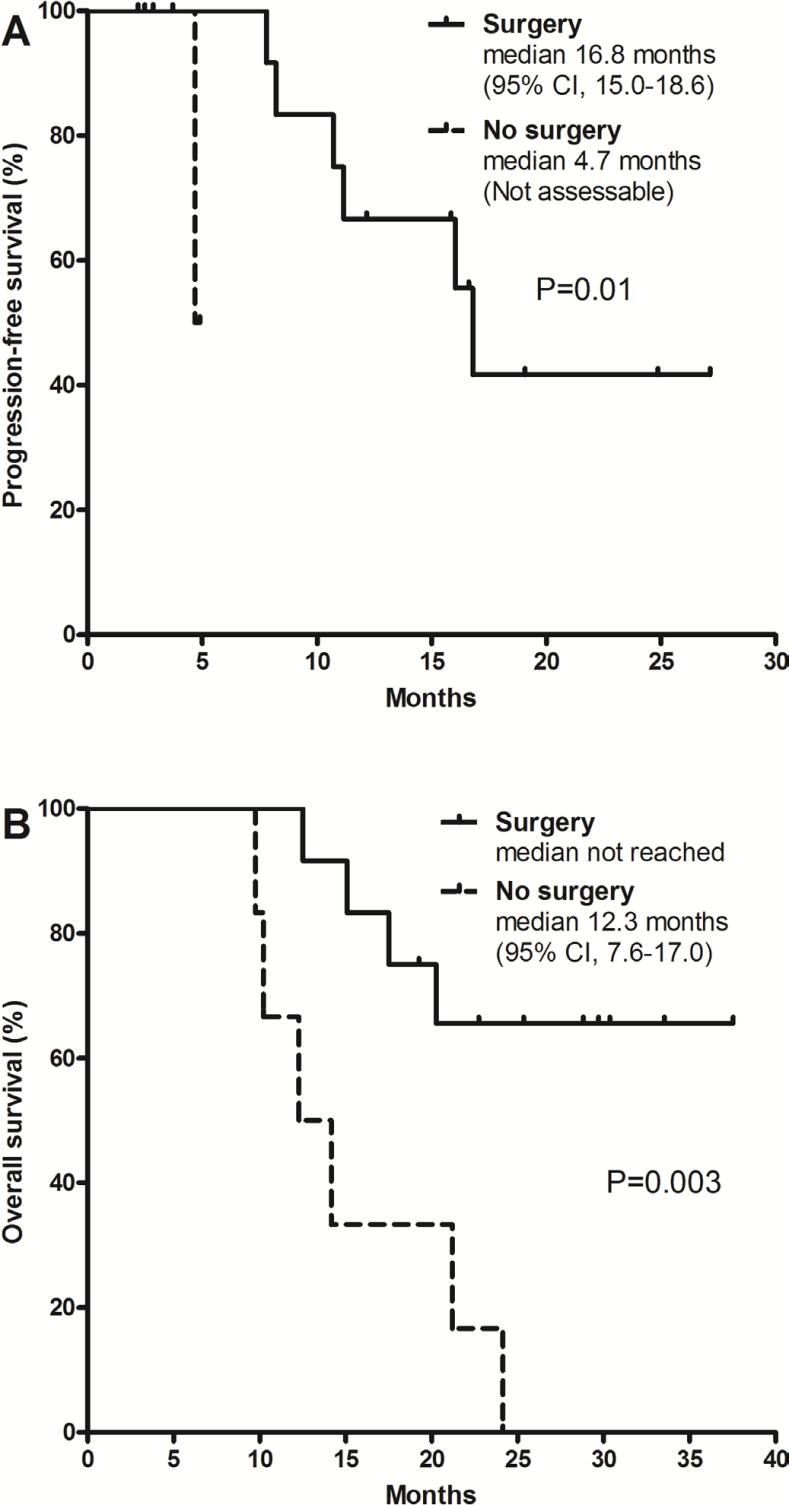
Progression-free survival **(A)** and overall survival **(B)** according to surgical resection.

With a median follow-up duration of 24.1 months (range, 14.1–32.3) for surviving patients, the 1-year progression-free survival (PFS) rate was 62% (95% CI, 35%–88%) and the 2-year OS rate was 49% (95% CI, 26%–72%). Median PFS was 16.8 months (95% CI, 9.4–24.2) and median OS was 21.2 months (95% CI, 14.2–28.2; Figure 1). Patients who underwent surgery showed significantly better PFS (median 16.8 vs. 4.7 months; p = 0.01; Figure 2A) and OS (not reached vs. 12.3 months; p = 0.003; Figure 2B) compared to those who did not receive surgery.

### Comparison of efficacy with FDR-GEM plus CAP

There was no statistical difference in patient characteristics between patients who received FOLFIRINOX and those who received fixed-dose rate (FDR)-gemcitabine (GEM) plus capecitabine (CAP) (Supplementary Table 1). Surgical resection rates were similar between the FOLFIRINOX and FDR-GEM plus CAP groups (12 patients, 67% vs. 11 patients, 61%; p = 1.00). Among patients who underwent surgical resection, microscopic complete resection (R0) was achieved in 9 patients in each group (75% vs. 82%; p = 1.00).

**Figure 3:**
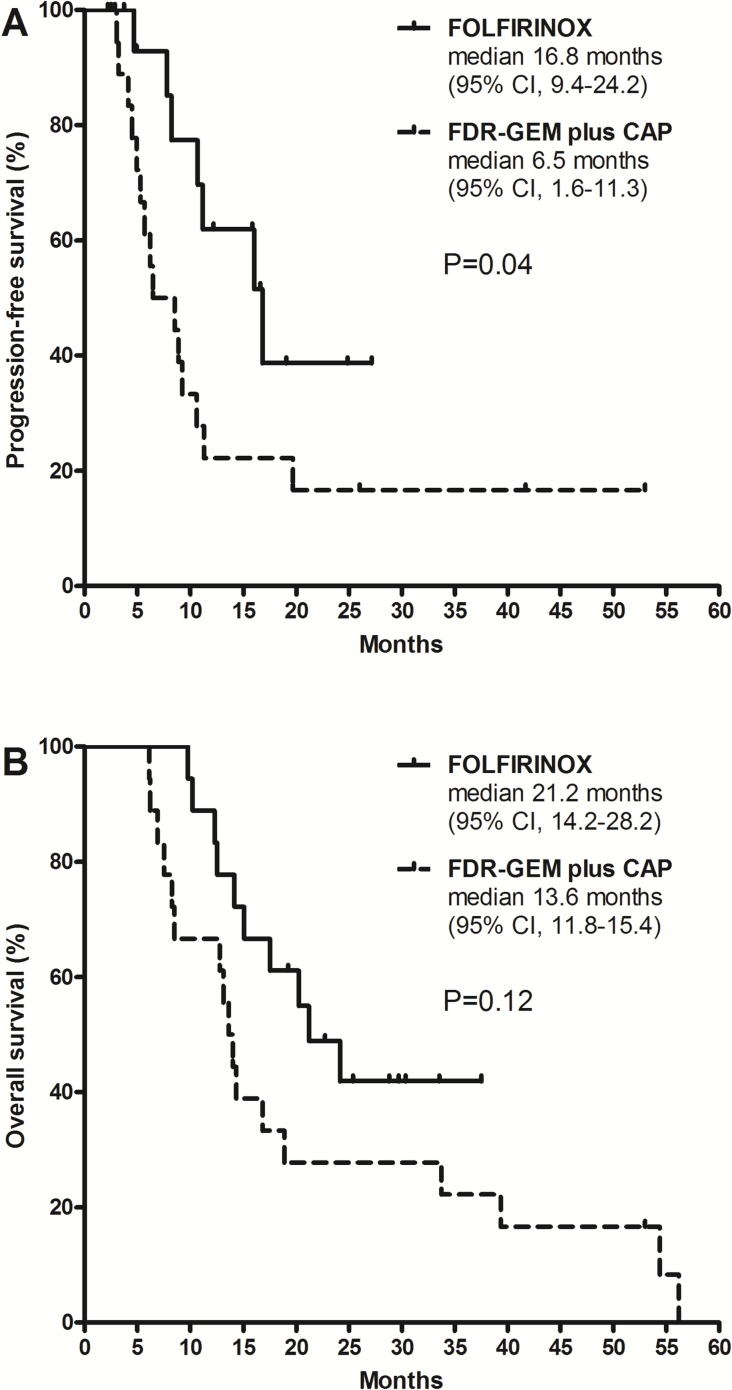
Progression-free survival **(A)** and overall survival **(B)** according to chemotherapy regimen (FOLFIRINOX vs. FDR-GEM plus CAP).

Patients who received FOLFIRINOX showed longer PFS compared to those who received FDR-GEM plus CAP (median 16.8 months [95% CI, 9.4–24.2] vs. 6.5 months [1.6–11.3]; p = 0.04) and this finding was statistically significant (Figure 3A). There was a trend toward improved OS in patients receiving FOLFIRINOX (median 21.2 months [95% CI, 14.2–28.2]) compared to those receiving FDR-GEM plus CAP (13.6 months [11.8–15.4]; p = 0.12; Figure 3B). Survival curves comparing PFS and OS between the two regimens are presented according to the status of surgical resection in Supplementary Figures (Supplementary Figure 1 and 2). Although the sample sizes were small and statistical significance was not present, FOLFIRINOX was consistently superior when patients were stratified according to the status of surgical resection.

### Multivariate analysis in pooled study population

Multivariate analyses for PFS and OS were performed with inclusion of all patients treated with FOLFIRINOX or FDR-GEM plus CAP (Table 3). FOLFIRINOX maintained its significance for better PFS in the multivariate model for PFS (vs. FDR-GEM plus CAP, hazard ratio [HR] = 0.27, 95% CI 0.09–0.81; p = 0.02) as well as age (<60 years vs. ≥60 years, HR = 0.29, 95% CI 0.10–0.86; p = 0.03), baseline CA 19-9 (elevated vs. normal, HR = 19.8, 95% CI 1.92–203.84; p = 0.01), and surgical resection (vs. no resection, HR = 0.05, 95% CI 0.01–0.21; p < 0.001). In the multivariate model for OS, baseline CA 19-9 within the normal range (vs. elevated, HR = 3.20 95% CI 1.11–9.19; p = 0.03) and surgical resection (vs. no resection, HR = 0.15, 95% CI 0.06–0.37; p < 0.001) were associated with superior OS.

**Table 3:** Multivariate analysis for PFS and OS in pooled study population treated with FOLFIRINOX and FDR-GEM plus CAP

PFS	Univariate analysis, HR (95% CI)	P	Multivariate analysis, HR (95% CI)	P
**Regimen**FOLFIRINOX vs.FDR-GEM plus CAP	0.41 (0.16–1.00)	0.04	0.27 (0.09–0.81)	0.02
**Age**≥60 vs. <60 years	0.92 (0.39–2.15)	0.84	0.29 (0.10–0.86)	0.03
**Baseline CA 19-9**≥UNL vs. <UNL	4.83 (0.65–36.01)	0.13	19.8 (1.92–203.84)	0.01
**Sex**Female vs. male	1.13 (0.48–2.63)	0.78	2.60 (0.95–7.11)	0.06
**Surgery**Yes vs. no	0.10 (0.03–0.31)	<0.001	0.05 (0.01–0.21)	<0.001
**OS**	**Univariate analysis, HR (95% CI)**	**P**	**Multivariate analysis, HR (95% CI)**	**P**
**Regimen**FOLFIRINOX vs.FDR-GEM plus CAP	0.56 (0.25–1.29)	0.12		
**Age**≥60 vs. <60 years	1.17 (0.54–2.53)	0.70		
**Baseline CA 19-9**≥UNL vs. <UNL	1.58 (0.59–4.25)	0.37	3.20 (1.11–9.19)	0.03
**Sex**Female vs. male	0.76 (0.34–1.71)	0.51		
**Surgery**Yes vs. no	0.22 (0.10–0.52)	<0.001	0.15 (0.06–0.37)	<0.001

PFS: progression-free survival; OS: overall survival; HR: hazard ratio; CI: confidence interval; FDR, fixed-dose rate; GEM, gemcitabine; CAP, capecitabine; UNL: upper normal limit.

## DISCUSSION

The current study revealed that FOLFIRINOX is well tolerated and effective in patients with BRPC. Exploratory analyses including the study population enrolled in our previous trial showed that FOLFIRINOX may provide better survival outcomes compared with a gemcitabine-based regimen, despite similar surgical resection rates.

In this study, neoadjuvant FOLFIRINOX resulted in an objective response rate of 33%, and surgical resection was performed in 67% of patients. R0 resection was achieved in half of all patients who received FOLFIRINOX, and median PFS and OS were 16.8 and 21.2 months, respectively. Surgical resection was associated with better PFS and OS, and in patients who underwent surgery, median PFS was 16.8 months and median OS was not reached with a median follow-up duration of 24.1 months. The results of the current study are in line with those of previous studies of FOLFIRINOX for LAPC. Although direct comparison is not possible because of the variation in the proportion of patients with BRPC was varied (17%–100%), previous studies have reported that surgical exploration rates and R0 resection rates ranged from 25%–83% and 18–67%, respectively, and median OS was reported to be 18–35 months [6, 11–17].

In the pooled analysis including BRPC patients from our previous study receiving the FDR-GEM plus CAP, FOLFIRINOX was significantly associated with better PFS (median 16.8 months vs. 6.5 months) and this remained significant in the multivariate model. Although there was no statistical significance, patients treated with FOLFIRINOX showed a trend toward better OS (median 21.2 months vs. 13.6 months). Our results are promising and warrant further investigation of FOLFIRINOX as neoadjuvant chemotherapy in patients with BRPC. One of the interesting findings is that survival outcomes seem to be enhanced with FOLFIRINOX despite similar rates of surgical resection between the two regimens (67% vs. 61%). This might indicate that the improved efficacy of FOLFIRINOX may be attributed to superior eradication of micrometastases rather than achievement of higher resection rates. Our results suggest that survival outcomes, such as PFS or OS, may be the appropriate endpoint for trials of neoadjuvant treatment in pancreatic cancer, considering that the resection rate is a subjective metric that reflects pretreatment staging and pre-/intraoperative decision making [3].

Surgical resection was the most powerful prognostic factor indicating favorable outcomes in the multivariate analysis. Therefore, surgery is recommended in patients for whom curative resection is technically possible and who are medically fit for surgery after neoadjuvant chemotherapy. In addition, elevated CA 19-9 was associated with poorer PFS and OS in BRPC patients, consistent with studies of patients with metastatic disease or following resection [18, 19]. This suggests that baseline CA 19-9 level might be a predictor of outcomes of neoadjuvant treatment in BRPC. Further studies with larger sample sizes are necessary to establish the predictive markers for the efficacy of neoadjuvant treatment in BRPC.

There were no unexpected toxicities of FOLFIRINOX in our study population. FOLFIRINOX treatment was feasible and most toxicities were successfully manageable with appropriate supportive management, as only one patient discontinued FOLFIRINOX due to treatment intolerance. However, grade 3 or 4 neutropenia developed in 83% of patients, and this resulted in frequent dose interruptions or modifications. The rates of severe neutropenia seemed to be much higher than those in a previous pivotal phase 3 trial of FOLFIRINOX for metastatic disease (46%) and recent patient-level meta-analyses of the regimen for LAPC (27%) [7, 20]. However, none of the patients in our study received primary G-CSF prophylaxis, in contrast to most previous studies testing FOLFIRINOX, which permitted primary G-CSF prophylaxis. Interestingly, a previous Japanese phase 2 trial of FOLFIRINOX in metastatic pancreatic cancer also reported a 78% rate of grade 3–4 neutropenia, similar to our results [21]. These suggest that full-dose FOLFIRINOX without primary G-CSF prophylaxis is associated with frequent severe neutropenia, at least in Asian patient populations. Considering that frequent dose interruptions or modifications result in low dose intensities that may lead to inferior efficacy, modified doses of FOLFIRINOX should be considered for improved tolerability and the maintenance of dose intensity. This is supported by the recent prospective trial which showed that modified FOLFIRINOX with primary G-CSF prophylaxis improved tolerability while maintaining efficacy [22].

Despite the promising efficacy of FOLFIRINOX in LAPC including BRPC, many elements of the neoadjuvant treatment regimen for BRPC remain to be optimized. Currently, the optimal number of cycles of preoperative FOLFIRINOX, the regimen for postoperative chemotherapy, and the incorporation of radiotherapy need to be defined in future prospective trials.

The current study has several caveats because of the nature of its retrospective design. Comparative analysis using pooled data is based on separate cohorts that may have different characteristics, although there were no significant differences among baseline characteristics. Moreover, the sample size is small for the derivation of definitive conclusions. However, the current analysis, using patient-level data, could contribute the insight that FOLFIRINOX may provide enhanced efficacy for patients with BRPC compared with a gemcitabine-based regimen. Despite the limitations of exploratory analysis, the results of the current study may be valuable because the strengths and weaknesses of FOLFIRINOX could be assessed by comparison to a gemcitabine-based regimen exclusively in patients with BRPC.

In conclusion, FOLFIRINOX is feasible and effective as neoadjuvant treatment for patients with BRPC. Based on this study, a prospective phase 2 trial of neoadjuvant chemotherapy consisting of 8 cycles of preoperative modified FOLFIRINOX and 6 cycles of postoperative gemcitabine is ongoing for patients with BRPC (NCT02749136).

## MATERIALS AND METHODS

### Patients

Patients with histologically documented pancreatic adenocarcinoma who received FOLFIRINOX at Asan Medical Center, Seoul, Korea between February 2013 and December 2014 were identified and their medical records were retrospectively reviewed. Among 86 patients who were treated with FOLFIRINOX, 18 patients (21%) were categorized as having BRPC according to the NCCN resectability criteria, without evidence of distant metastasis (Supplementary Methods) [4]. The Institutional Review Board of Asan Medical Center approved this study and waived the requirement for informed consent.

### Treatment

Neoadjuvant FOLFIRINOX consisted of a 2-hour intravenous infusion of oxaliplatin 85 mg/m^2^ followed by a 90-min intravenous infusion of irinotecan 180 mg/m^2^ and a 2-hour infusion of leucovorin 400 mg/m^2^, followed by an intravenous bolus of 5-FU 400 mg/m^2^ and a 46-hour continuous infusion of 5-FU 2,400 mg/m^2^, administered every 2 weeks, as described in the PRODIGE 4 trial [7]. Subsequent dose modification or delays for adverse events were guided based on the specified protocol [7]. The timing of surgical resection and the postoperative treatment were determined following discussion at the multidisciplinary team clinic.

### Study assessments

Baseline assessments include medical history, physical examination, complete blood counts, serum chemistry and electrolytes, CA 19-9, triphasic computed tomography scan, magnetic resonance imaging, and ^18^F-FDG-positron emission tomography- computed tomography scan. Tumor responses were assessed after every 3 or 4 cycles of FOLFIRINOX and graded according to the Response Evaluation Criteria in Solid Tumor, version 1.1 [23]. Toxicities were graded according to the National Cancer Institute Common Terminology Criteria for Adverse Events, version 4.03. Serum CA 19-9 was measured at baseline and at every imaging follow-up.

### Comparison of efficacy with FDR-GEM plus CAP

Our group previously performed a phase 2 study of FDR-GEM plus CAP for borderline resectable or unresectable locally advanced pancreatic adenocarcinoma, and the results of this study were published elsewhere [9]. In brief, patients received 3 to 6 cycles of FDR-GEM plus CAP, consisting of intravenous gemcitabine at 1,250 mg/m^2^ with a 10 mg/m^2^/min infusion rate on days 1 and 8, and oral capecitabine at 950 mg/m^2^ twice daily on days 1–14, every 3 weeks. Among 43 patients included in this trial, 18 patients were prospectively classified as BRPC according to the NCCN resectability criteria. The data of these patients were pooled with those of the current study population treated with FOLFIRINOX for the comparison analysis of the efficacy between the two regimens (18 patients each in the two groups).

### Statistical analysis

PFS was defined as the time from the initiation of chemotherapy to the date of disease progression, recurrence after surgery, or death, whichever occurred first. OS was defined as the time between the initiation of chemotherapy and any cause of death. Categorical variables were compared using chi-square or Fisher's exact tests, as appropriate. OS and PFS curves were estimated by the Kaplan-Meier method and compared by log-rank tests. Multivariate analyses for PFS and OS were based on the pooled analysis including all BRPC patients treated with either FOLFIRINOX or FDR-GEM plus CAP. All statistical analyses were performed using the Statistical Package for the Social Sciences (IBM SPSS, Chicago, IL, USA) version 21.0, and all tests were two-sided with 5% defined as the level of significance.

## SUPPLEMENTARY MATERIALS FIGURES AND TABLE



## Abbreviations

BRPC: borderline resectable pancreatic cancer
CA 19-9: cancer antigen 19-9
CAP: capecitabine
CCRT: concurrent chemoradiotherapy
CI: confidence interval
FDR: fixed-dose rate
FOLFIRINOX: fluorouracil(5-FU), leucovorin, irinotecan, and oxaliplatin
G-CSF: granulocyte-colony stimulating factor
GEM: gemcitabine
HR: hazard ratio
LAPC: Locally advanced, non-metastatic pancreatic cancer
NCCN: National Comprehensive Cancer Network
OS: overall survival
PFS: progression-free survival
RECIST: Response Evaluation Criteria In Solid Tumors
RDI: relative dose intensity.
