# Supplementary material for: Efficacy and safety of neoadjuvant FOLFIRINOX for borderline resectable pancreatic adenocarcinoma: improved efficacy compared with gemcitabine-based regimen
Source: Oncotarget. 2017 May 16;8(28):46337–47. doi: 10.18632/oncotarget.17940 (PMC5542271; doi:10.18632/oncotarget.17940)
Supplement: Supplementary file 1 [file oncotarget-08-46337-s001.pdf]

Efficacy and safety of neoadjuvant FOLFIRINOX for borderline resectable pancreatic adenocarcinoma: improved efficacy compared with gemcitabine-based regimen

SUPPLEMENTARY METHODS

Definition of borderline resectable pancreatic cancer (BRPC) according to the NCCN resectability criteria

According to the NCCN resectability criteria, tumors considered BRPC include the following: (1) venous contact with the superior mesenteric vein (SMV) or portal vein (PV) of greater than 180°, contact of 180° or lesser with contour irregularity of the vein or thrombosis of the vein but with suitable vessel proximal and distal to the site of involvement allowing for safe and complete resection and vein reconstruction, contact with the inferior vena cava; (2) arterial contact with common hepatic artery (CHA) without extension to celiac axis or hepatic artery bifurcation allowing for safe and complete resection and reconstruction, solid tumor contact with the superior mesenteric artery of 180° or lesser or presence of variant arterial anatomy (ex: accessory right hepatic artery, replaced right hepatic artery, replaced CHA and the origin of replaced or accessory artery) and the presence and degree of tumor contact should be noted if present as it may after surgical planning for pancreatic head/uncinate process cancer; and (3) arterial contact with the celiac axis (CA) of 180° or lesser or contact with the CA of greater than 180° without involvement of the aorta and with intact and uninvolved gastroduodenal artery for pancreatic body/tail cancer.

SUPPLEMENTARY FIGURES AND TABLE

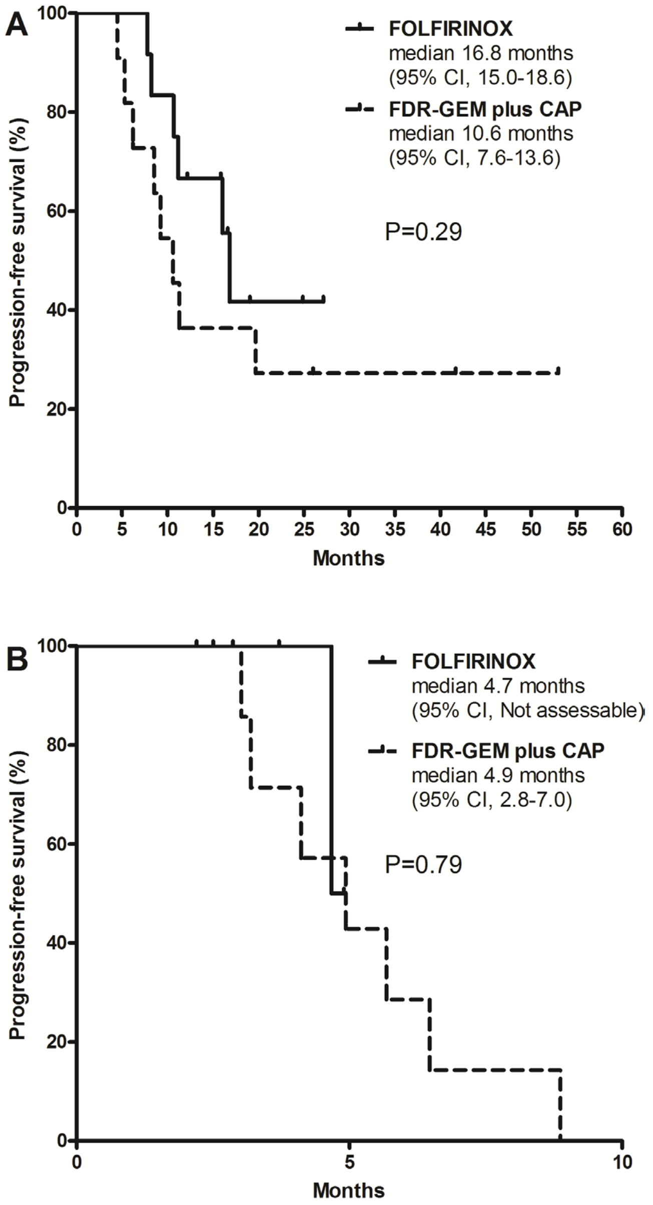

Supplementary Figure 1: Comparison of progression-free survival between FOLFIRINOX and FDR-GEM plus CAP according to surgical resection status **(A)** Surgical group **(B)** Non-surgical group.

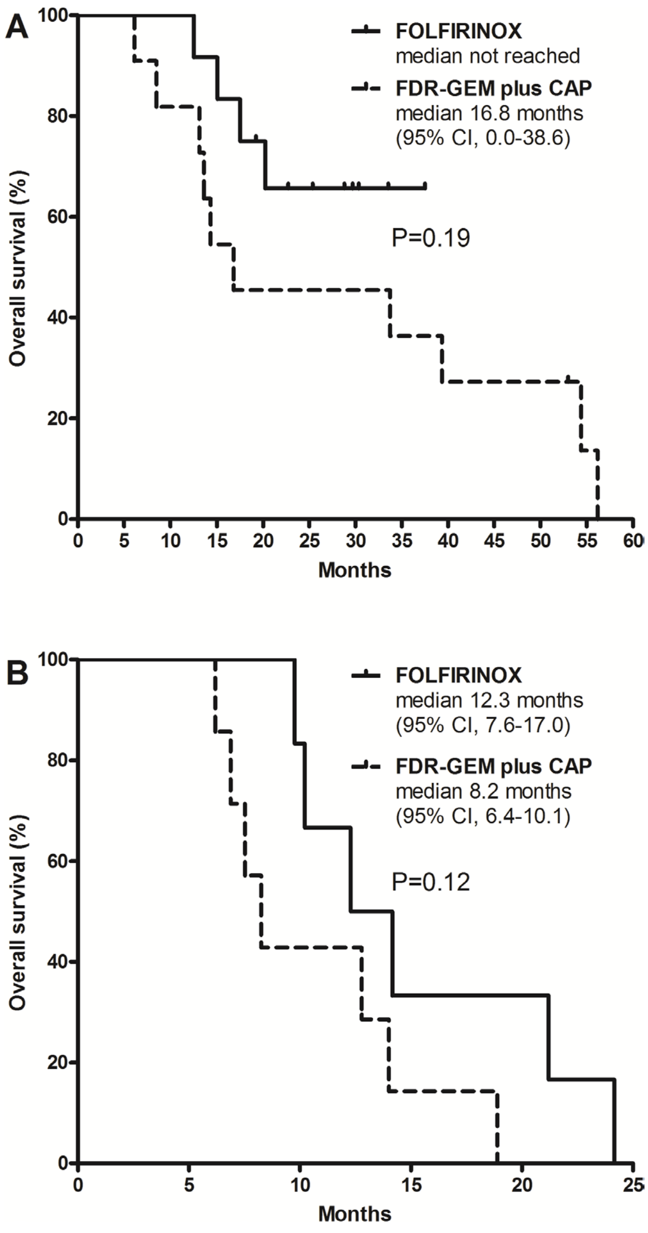

Supplementary Figure 2: Comparison of overall survival between FOLFIRINOX and FDR-GEM plus CAP according to surgical resection status **(A)** Surgical group **(B)** Non-surgical group.

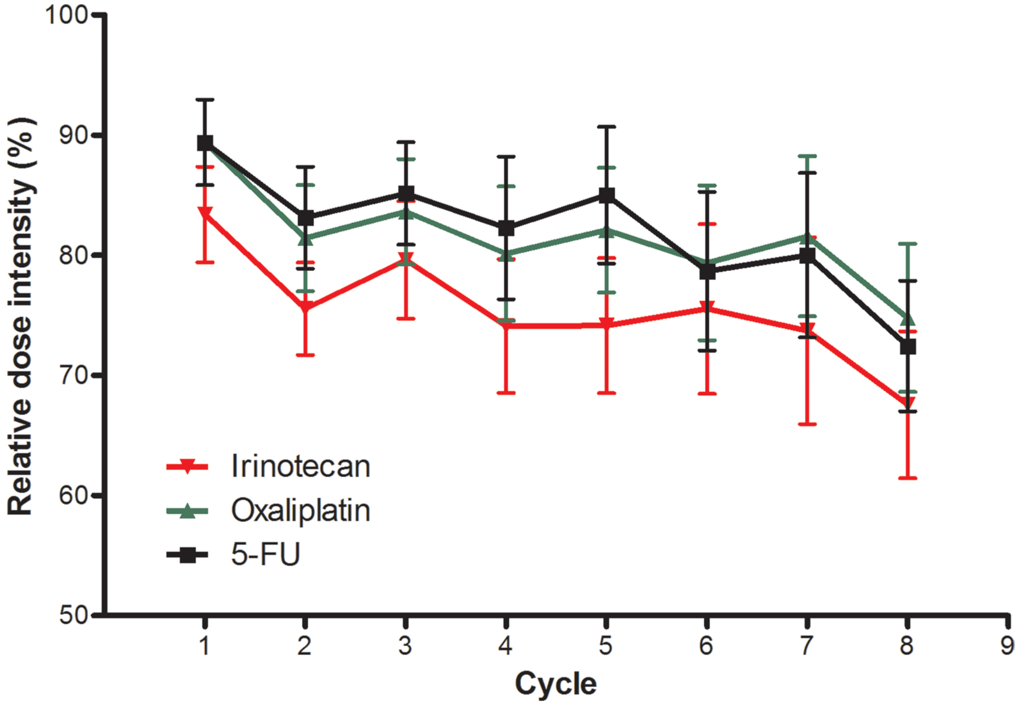

Supplementary Figure 3: Relative dose intensity of FOLFIRINOX.

Supplementary Table 1: Baseline characteristics

	FOLFIRINOX (n = 18)	FDR-GEM plus CAP (n = 18)	P values
**Sex**			0.50
Male	9 (50%)	12 (67%)	
Female	9 (50%)	6 (33%)	
**Age**	54 (range, 29–73)	62 (range, 44–72)	0.76
**Primary tumor site**			0.73
Head	10 (55%)	13 (72%)	
Body or tail	7 (39%)	4 (22%)	
Multicentric	1 (6%)	1 (6%)	
**ECOG PS**			0.74
0	8 (44%)	9 (50%)	
1	10 (56%)	9 (50%)	
**Baseline CA 19-9**			0.81
Normal	4 (22%)	3 (17%)	
>1 and <2 x UNL	4 (22%)	3 (17%)	
>2 x UNL	10 (56%)	12 (66%)	
**Response**			1.00
Partial response	6 (33%)	5(28%)	
Stable disease	12 (67%)	12 (67%)	
Progressive disease	0	1 (6%)	
**Regional lymph node metastasis**	10 (56%)	11 (61%)	1.00
**Surgery**	12 (67%)	11 (61%)	1.00
R0	9 (75%)	9 (82%)	1.00
R1	3 (25%)	2 (18%)	
**Postoperative treatment**			0.75
No	2 (17%)	2 (18%)	
Chemotherapy only	7 (58%)	8 (73%)	
CCRT only	2 (17%)	0	
CCRT and chemotherapy	1 (8%)	1 (9%)	
**Postoperative chemotherapy regimen**			
Gemcitabine	3 (38%)	1 (11%)	
FOLFIRINOX	5 (62%)	0	
FDR-GEM plus CAP	0	8 (89%)	

PR = partial response, SD = stable disease, PD = progressive disease, CA 19-9 = cancer antigen 19-9, UNL = upper normal limit, CCRT = concurrent chemoradiotherapy.
